# Linguistic mechanisms of knowledge-exchange in a dark-web money laundering forum

**DOI:** 10.1371/journal.pone.0329777

**Published:** 2025-08-05

**Authors:** Emily Chiang

**Affiliations:** Aston Institute for Forensic Linguistics, Aston University, Birmingham, United Kingdom; Paris 1 University: Universite Paris 1 Pantheon-Sorbonne, FRANCE

## Abstract

Money laundering facilitates serious crime, enables the expansion of criminal operations, and destabilises economies. Extant scholarship is largely concerned with anti-money laundering approaches, with far less attention being paid to the language and behaviours of the individuals who engage in money laundering. ‘Dark-web’ discussion fora are prime loci for illicit knowledge exchange and key enablers of money laundering, yet, are underexplored as sites for understanding the online activities and behaviours of users. This paper reports on a corpus-assisted discourse analysis of one such forum, guided by research questions around the key topics and common linguistic strategies by which knowledge is exchanged within a large community of individuals interested in money laundering, and the ways in which this community serves its members. The analysis identifies the forum as an extremely efficient and productive site for knowledge-exchange and thus ‘criminal upskilling’, which is attributed to three core characteristics: a strict adherence to community rules, a highly knowledgeable user base, and a culture of friendliness and reciprocity.

## Introduction

Money laundering (ML) or the “false representation of criminal earnings as legitimate earnings” [1: p.163] essentially involves disguising the origins of ill-gotten proceeds through complex financial systems. Crucially, this practice enables the continuation and expansion of criminal operations of various kinds, and works to destabilise financial systems and economies. Broad legal definitions of ML coupled with the occluded nature of the predicate crimes that produce illegitimate proceeds makes measuring the magnitude of the problem extremely difficult [1,2], although the number of USD laundered globally per year reportedly runs into the trillions [3]. Furthermore, the range and seriousness of the predicate crimes that rely on or benefit considerably from the practice of ML e.g. the trade of illicit drugs and arms, fraud, environmental crimes, human trafficking and modern slavery, etc. imply an untenable human cost.

While research in this area is abundant, studies exploring the individual and group-level behaviours surrounding ML activity are rare. One important issue concerns clandestine online platforms that are unindexed by mainstream search engines and offer perceived anonymity, enabling ‘free’ discussion and advice-sharing around illicit and illegal activities. While general discussions around the nature of money laundering occur on clear web discussion fora e.g. various subreddits, such ‘dark-web’ discussion fora have been identified as key enablers of ML and fraud [1]. Yet, we do not have a clear understanding of the interaction and activity that occurs in such spaces, or how they might support individuals hoping to become successful launderers. Responding to this gap, this paper reports on a linguistic analysis of the discursive practices and interactional behaviours observed in group conversations within a dark-web ML-focused forum known as/d/Laundromat. The broad aim is to unpick the linguistically enabled mechanisms of knowledge-exchange in this online learning environment as a route to better understanding how dark-web spaces can facilitate ML.

### Key areas of interest in money laundering research

Researchers have, over the past few decades, sought to better understand the nature, processes and global impact of ML. Tiwari et al. [4] identify six key areas of interest across the ML literature, which are briefly summarised here before the linguistic research is discussed.

Tiwari et al. [4] note a significant portion of research concerning anti-money laundering (AML), including work on the effectiveness of global AML legislation (e.g. [5,6]), which highlights a need for greater international cooperation [7]. The policing of ML has increasingly been pushed onto financial institutions themselves, who are often required by law (e.g. the US Bank Secrecy Act of 1970, the UK Proceeds of Crime Act 2002) to report suspicious activity and hand over relevant documentation to regulating bodies [1]. Overall, there are mixed results regarding the effectiveness of AML regulations [8], and despite a steep rise in AML laws, regulations and policing over recent decades, there has been no observable reduction in the prevalence or costs associated with ML [1]. Tiwari et al. [4] also discuss automated ML-detection systems based on both statistical (e.g. [9]) and machine-learning (e.g. [10]) methods, noting in particular their overall scarcity in comparison with fraud-detection tools.

Another key area of research explores the relationship between ML and tax havens (e.g. [11]), offshore financial centres [12] and accounting practices [13] as well the effects of ML on the economy overall. Broadly, this work shows that ML harms the economy through monetary and socio-economic instability, and by promoting corruption and making financial systems more vulnerable [14,15]. ML is also shown to increase criminal activity and the shadow economy, and to reduce tax collections [16].

Tiwari et al. [4] also note the importance of actors and their various roles in understanding motivations for committing financial crime. Research shows, for example, that criminals often launder their own money rather than seeking professional assistance [17] but also that finance professionals (accountants, auditors etc.) can be integral to criminal networks [18]. While a clear motivation for engagement in ML is the financial incentive [19], the decision to commit financial offences is also influenced by individuals’ occupational roles, social relationships and dynamics, situational circumstances, and organisational cultures [20,21].

Understanding the magnitude of the problem involves determining both the extent to which money is laundered and the amount of money that gets laundered [3,22,23] as well as evaluating the methods used to measure and derive estimated figures (e.g. case studies, proxy variables and economic models). One of the most widely cited figures comes from Walker [22], whose model estimated that $US2.85bn per year is laundered globally, while Schneider and Barone [3] offer a more recent estimate of US$3.345tn. However, the clandestine nature of the crimes underlying ML activity mean that precise figures are virtually unobtainable [2], and as such, the accuracy of these estimates remains contested [4].

Another important line of research has been driven by technological advancements over recent decades that have presented new opportunities and methods for ML. Perhaps the most impactful of these is the increased opportunity for connectivity and even socialisation in online spaces between individuals with illicit and criminal interests. An increase in global collaboration between perpetrators across various interactional contexts has led to an increase in opportunities for illicit activity [24], as well as new avenues for ML, for example, through online gaming [25]. There has also been interest in the use of cryptocurrencies in ML, given their lack of regulatory oversight and their capacity (in some cases) to facilitate anonymous transactions [4]. However, Dostov and Shust [26] note that their volatility, lack of universal acceptability and the inevitable requirement to exchange them for fiat money make cryptocurrencies less attractive. This is supported by the observation that cash remains the primary commodity used in ML schemes, largely due to the difficulties associated with tracing its physical movement [1,27]).

Where research interests have focused on AML methods, scale and impact, actors’ motivations and technological developments, little attention has been paid to the linguistic activities and behaviours around ML. Having said this, a small number of studies demonstrating the applicability of linguistic frameworks in this context are worth noting (e.g. [28,29]). Hourani-Martín & Tabares-Plasencia [29] examined verb-noun constructions in a corpus of Spanish legal texts, finding that variation in ML-related terminology across Hispanic states is common, despite efforts to homogenise the language used in this domain. Shuy [28], on the other hand, analysed the language of an undercover DEA agent tasked with eliciting information of evidentiary value from a suspect in an ML investigation. From covert tape recordings of interviews between the agent and suspect, Shuy demonstrated the use of linguistic concepts such as speech acts [30] and topic management to address questions around the suspect’s potential self-incrimination. But beyond this work, linguistic research into ML is extremely limited. In particular, there is a distinct lack of focus on the individuals and groups that engage in ML, and their linguistic behaviours and activities. It is widely understood that dark-web fora facilitate myriad criminal activities, in part through the provision of illicit marketplaces as well as the opportunity for community-building amongst anonymous users with shared deviant interests [31,32] Those focused specifically on the exchange of knowledge around ML constitute rich sites of linguistic activity that offer a unique window onto the practices and behaviours of people interested in learning about ML, and in turn, crucial insights for developing effective countermeasures. They are also vastly under-explored, despite being recognised as a key enabler of the crime [1,4].

### Forensic linguistic research on online criminal communities

As an applied academic discipline, forensic linguistics is chiefly concerned with the analysis of language in criminal and legal contexts for the purpose of improving the delivery of justice. Increasingly within this field, the phenomenon of online community-building around specific criminal activities and harmful behaviours has been brought into focus, and researchers have begun to demonstrate the use of linguistic analytical methods to shed light on the nature of these communities (e.g. [32–35]). Chiang et al. [34] and Chiang [32] for example, examine the rhetorical moves [36] used by members of dark-web communities formed around an interest in child sexual exploitation and abuse (CSEA). In a forum focused on the exchange of abuse imagery, Chiang et al. [34] observed that common moves included rapport building and demonstrating appreciation for another user’s uploaded imagery. The authors also noted that direct requests for particular types of images were typically unsuccessful and likely to prompt some kind of reprimand from other forum users. Chiang [32] examined forum posts authored by newcomers requesting membership into established CSEA communities, finding linguistic expressions of competence regarding CSEA offending behaviours to be a key aspect of these attempts. In the same domain, MacLeod and Grant [37] and Grant and MacLeod [33] demonstrated how understanding around the linguistic construction of identity can inform the training of undercover police officers required to impersonate abusers and victims online. Booth [35] on the other hand, examined linguistic identity in an online community of far-right extremists. Using methods from corpus linguistics (the software-assisted analysis of large bodies of machine-readable texts) and discourse analysis, she observed that group members were not as ideologically homogenous as previous scholarship would suggest.

A common idea throughout much of this research is that harmful online communities often operate as ‘communities of practice’; a concept developed within education research in the 1990s that sought to describe social systems for learning (see [38,39] Rather than being defined by geographical location or demographic characteristics, communities of practice (CoPs) are established on the basis of ongoing engagement in relation to specific interests and endeavours [40]. As such, the concept provides a useful lens for examining groups oriented to “learning in the wild” [41: p.631], i.e., self-organising online groups seeking to learn outside of formal learning institutions. Given the lack of formal, institution-based learning environments for those whose interests are socially harmful, illicit and even criminal, the community of practice as a social learning system has a particular relevance regarding groups who frequent dark-web fora focused on, for example, CSEA, fraud, or indeed money laundering. While research on online criminal communities frequently acknowledges that online platforms exist at least in part as a way for group members to learn from each other via the exchange of tips, advice and emotional support regarding offending [34,42,43]; questions around the specific linguistic means by which knowledge is exchanged in these environments, and how they support the upskilling of individuals across various criminal domains, have not yet been addressed. Building on previous work in forensic linguistics, this work seeks to address this gap and at the same time contribute new insights regarding a previously unexplored criminal community of practice.

### Objectives

This paper seeks to contribute a linguistically informed understanding of the nature and behaviours of a money laundering-focused dark-web community of practice by reporting on a corpus-assisted analysis of discursive practice in the forum known as/d/Laundromat. Its main objectives are to explore and describe the linguistic mechanisms of knowledge-exchange in/d/Laundromat, and to consider the role of this forum in supporting individuals’ learning regarding money laundering activity. It does this by identifying key topics of discussion and common linguistic constructions and discursive practices associated with knowledge-exchange using tools from corpus linguistics, including word frequency and keyword lists, n-grams, and concordance lines (see ‘Procedure’ section). It also draws on Biber and Barbieri’s [44] functional classification of ‘lexical bundles’ to interpret the discourse functions of common linguistic constructions in the forum posts. Research questions are summarised as follows:

RQ1. What are the key discussion topics in/d/Laundromat?

RQ2. What are the common linguistic constructions by which knowledge is exchanged?

RQ3. To what extent does this community of practice serve the needs of its members and how does it do this?

By addressing these questions, this paper empirically contributes to an overall understanding of the individual and group-level linguistic behaviours around the practice of ML, as well as a broader understanding of how a self-organising online community with a criminal focus engages in the practice of knowledge-exchange. By describing a previously unexplored dark-web community of practice, this work also hopes to develop further understanding around the role of dark-web spaces in supporting criminal upskilling and facilitating crime more generally.

## Methods

### The/d/Laundromat corpus

This study arises from a wider Innovate UK-funded project investigating the language of online fraud communities, for which ten dark-web fraud-related fora were scraped using the Tor browser and custom Python scripts developed by Forensic Pathways Ltd., a company specialising in technology for dark-web monitoring and investigation. The data for the current study comprises forum posts from the only one of these for a dedicated to discussions on ML, namely/d/Laundromat; a ‘subdread’, (i.e. a single-topic subforum) found on the dark-web forum known as Dread. Structured similarly to clear-web forum Reddit, Dread was created in 2018 with the express purpose of facilitating online community-building by groups interested in illicit marketplaces. Its scope has broadened over time and it now comprises c.1750 subdreads devoted to discussions on, for example, cybersecurity, cryptocurrencies and fraud, each with its own rules, tutorials, and moderators. At the time of data collection,/d/Laundromat had 1,849 active users. Users are expected to adhere to four central rules (taken verbatim from the/d/Laundromat Rules page):

1. No posts about unrelated topics
2. No promotion of services of any kind
3. Be normal, kind, and post real life things
4. No duplicate threads or spamming

Further to these rules, users are offered a narrow list of allowed topics including “tax evasion, tax havens, keeping your cryptocurrencies anonymous…” and directed to other subdreads that allow the promotion of services (e.g. offering to clean another user’s funds). As rule 3 demonstrates, there is an expectation of civility, and that all advice/information offered is supported by real world experience. The fourth rule reflects the community’s (or at least the moderators’) desire for ‘high quality’ posts and original content, which is further incentivised by a rewards program.

All/d/Laundromat posts between its inception in December 2018, and October 2022 were scraped for analysis. Following retrieval, the data was cleaned to remove ‘noise’, i.e. non-interactional content including URLs, html mark-up, numeric values like credit card numbers, and automated bot posts. This was carried out by Forensic Pathways through an interative process involving the use of a feature extractor that assigned numerical scores to posts based on the relative amount of list items of irrelevant content and a support vector classifier trained on the extracted features. The resulting dataset features only forum users’ post content and consists of 6,546 forum posts within 843 individual discussion threads, totalling 378,859 tokens (words).

### Procedure

**Fig 1:**
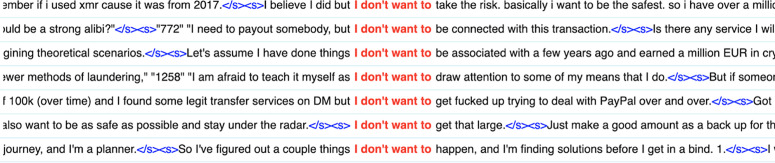
Example concordance lines for the 5-gram *I don’t want to.*

The data was interrogated using Antconc [45] and Sketch Engine [46]; two tool suites offering various functions for corpus linguistic analysis, i.e. the automated analysis of large bodies of machine-readable text. To begin, word frequency and keyword lists were generated. Word frequency lists display all words found in a corpus according to their frequency, whereas key word lists present words ranked by their ‘keyness’, or noteworthiness, as statistically determined in relation to a large, general reference corpus [47] in this case, English Web 2021. Word and keyword lists offer general information regarding the “aboutness” of the corpus in question [48] and therefore offer useful insights regarding the prominent topics of discussion for/d/Laundromat users. The top 25 lexical items (i.e. words that carry semantic information such as nouns, verbs, adjectives, and adverbs) from each list were selected for closer analysis and categorised into parts of speech, and according to the broader topics they point to. Grammatical function words like *the*, *and*, *to*, etc. were discounted. Following this, a word-level n-gram analysis was conducted, by which the most common five-word sequences (*5-grams*) were identified and examined with the aim of determining common discursive practices as well as gathering further information regarding discussion topics. 5-grams were selected for the amount of information they carry coupled with their lower frequency across the dataset in comparison to shorter sequences such as 3-grams or 4-grams. This allowed for a detailed, qualitative examination of every instance of each of the 20 most common 5-grams in its local context using concordance lines, of which there are 263 in total. Concordance analysis allows the researcher to examine the n-gram of interest as it appears in relation to what was written immediately before and after, in order to understand the functions and topics associated with that n-gram. Fig 1 provides an illustrative example of concordance lines for the common 5-gram *I don’t want to*. It is worth noting that contractions (e.g. *don’t*, *can’t*) here are treated as two individual tokens, and so phrases like *you don’t want to* or *i don’t know how* are considered 5-grams.

From the context derived through concordance analysis, the 5-grams were classified according to the broad discursive functions that they were observed to work towards, and then interpreted according to Biber and Barbieri’s [44] functional taxonomy of “lexical bundle” types (p. 264). The authors use ‘lexical bundle’ to refer to high-frequency multi-word sequences (such as 5-grams) that are typically structurally incomplete and without idiomatic meaning, but serve important discourse functions. From an investigation of several spoken and written registers within a university through the analysis of, for example, classroom discourse, text books, student/advisor meetings, and student study groups, Biber and Barbieri [44] identified three types of bundle, each with associated functional subtypes:

1. *Stance bundles* express epistemic evaluations and attitudinal meanings. They can express *epistemic* stances around the speaker’s certainty of a proposition (e.g. *I don’t know what* we’re doing later), stances of *desire* (e.g. *I don’t want to* do it), of *obligation* (e.g. *you just have to finish* this one), of intention or prediction (e.g. *what we’re going to* do is) and of *ability* (e.g. I should *be able to do that* tomorrow).
2. *Discourse organising bundles* indicate the structure of the discourse, through *topic introduction* (e.g. *What we should do is* try again tomorrow), *topic elaboration* and *clarification* (e.g. this talk *is all about the ways* we), and *identification* and/or *focus* (e.g. *one of the reasons we* do this is).
3. *Referential bundles* point to a particular entity or attribute as being important. This includes bundles that express *imprecision* (e.g. *or something like that*), that *specify attributes* (e.g. *the sort of thing* we are talking about) or indicate *time*, *place* or *text-deixis* (e.g. *at the end of the* show, *as seen in the* data).

Because these bundle types and their associated sub-functions are derived from an analysis of largely pedagogical registers, they form a useful framework for examining the functions of frequent 5-grams in a knowledge-exchange online platform such/d/Laundromat, and addressing questions around linguistic mechanisms of knowledge exchange in online communities of practice.

### Limitations

An inherent difficulty with any dark-web research is that sites often have a short lifespan, and can disappear and reappear after days or months, sometimes under a new name or in some other incarnation. As such, even the most comprehensive data scraping may not capture full accounts of all the interaction that has taken place on a particular forum. Furthermore, being limited to a single forum, the findings from this work are specific to the community in question and cannot be generalised. However, this modest sized corpus spans almost two years of interaction and nevertheless provides a unique insight into the linguistic behaviours of a large group of individuals brought together by an interest in money laundering, and as such, offers a rare look at the inner workings of an ML-focused community of practice. In doing so, it provides a background of understanding regarding one type of criminal online community against which research on other communities might be compared and interpreted.

### Ethics

This research was carried out in accordance with guidelines set out by the Aston University Research Integrity and Ethics Committee (URIEC) as part of a wider Innovate UK-funded project. All data was obtained from publicly accessible online fora which did not require the creation of user accounts, and stored securely on password-protected computers in accordance with a data sharing agreement with Forensic Pathways. The data is naturally pseudonymous in that only screen names are used and these are not linked to personally identifying information. No screen names are included in the textual examples given in this paper or any other form of research dissemination. Researchers exposed to the data had continuous access to psychological support provided by the Aston Institute for Forensic Linguistics.

## Analysis

### Prominent topics

Prominent discussion topics were derived in part from the 25 most common lexical items in the corpus (displayed in Table 1).

**Table 1:** 25 most frequent lexical items in the Laundromat corpus.

Word	Raw frequency
*cash*	2232
*money*	2229
*do*	1945
*get*	1469
*crypto*	1267
*account*	1184
*bank*	1118
*need*	1079
*business*	1009
*btc*	948
*know*	918
*use*	910
*make*	868
*buy*	833
*want*	811
*people*	799
*good*	704
*xmr*	666
*go*	648
*pay*	645
*exchange*	612
*time*	611
*sell*	608
*clean*	578
*work*	576

The list largely comprises nouns and lexical verbs (i.e. verbs that carry semantic meaning as opposed to grammatical function), including several that point broadly to the topics of finance and business (e.g. *cash, money, crypto, account, bank, business, btc, xmr, exchange, buy, pay, sell*). The presence of the terms *btc* (Bitcoin) and *xmr* (Monero) indicates the significance of cryptocurrencies in discussions around money laundering, despite suggestions that their inherent volatility and associated practical difficulties makes them a less desirable asset than cash [26] and that cash remains the basis of most ML schemes [1]. The high frequency of references to Monero - a decentralised cryptocurrency designed to ensure the untraceability of financial transactions - points to a general interest among/d/Laundromat users in maintaining security and anonymity, which is unsurprising considering the illegality of the activity at the centre of these discussions.

Also worth noting is that just over half (14) of the high-use items are verbs, and of these, 11 can be classed as activity verbs, which refer to agentive activities carried out by an actor [49], i.e. *do, get, use, make, buy, go, pay, exchange, sell, clean, work*. The prevalence of terms relating to actions and processes reflects a strong focus on the approaches, methods, and practices involved in ML as topics of discussion. The presence of three mental verbs on this list (those reflecting a mental state [49]), i.e. *need*, *know* and *think*, does not signify particular topics, but hints at the discursive practices of requesting and sharing knowledge.

Keywords offer further insight into the topics that characterise/d/Laundromat discussions. The top 25 keywords observed are reported in Table 2.

**Table 2:** Top 25 keywords in the/d/Laundromat corpus.

Key word	Raw frequency	Relative frequency (per million words)	Keyness score
*xmr*	653	1430.60	1370.1
*monero*	527	1154.56	993.8
*btc*	931	2039.65	746.2
*launder*	590	1292.58	644.0
*crypto*	1278	2799.86	500.2
*localmonero*	175	383.39	384.1
*kyc*	245	536.75	372.7
*laundering*	402	880.71	226.0
*nft*	191	418.45	221.9
*cashout*	101	221.27	197.7
*legit*	326	714.21	194.0
*localbitcoin*	81	177.46	177.5
*coinbase*	121	265.09	175.7
*opsec*	70	153.36	144.5
*wallet*	575	1259.72	132.5
*paxful*	59	129.26	127.7
*cashapp*	55	120.49	119.3
*dnm*	54	118.30	113.7
*clearnet*	53	116.11	112.0
*darknet*	52	113.92	101.5
*bitcoin*	531	1163.32	101.3
*launderer*	50	109.54	98.8
*eth*	89	194.98	97.8
*nfts*	65	142.40	97.3
*fullz*	42	92.01	91.6

Further indicating the focus on cryptocurrencies in the forum discussions, just over half (13/25) of the keywords relate to this topic. These include names of currencies and other digital assets, (*xmr, monero, btc, nft, bitcoin, eth[ereum], nfts*), terms referring to cryptocurrency exchanges and management platforms, (*localmonero, localbitcoin, coinbase, wallet, paxful*) and one general, abbreviated term (*crypto*). Other terms that arise from the keywords list refer directly to the practice of ML itself, (*launder, laundering, launderer*), the dark-web context (*dnm (darknet market), darknet, clearnet*) and online fraud activity (*cashout, fullz, kyc*), the latter demonstrating a particularly close association between ML and fraud. Other terms include the name of a mobile payment service (*cashapp*), an abbreviated term for *operations/operational security* (*opsec*), and an abbreviated form of *legitimate* (*legit*). A review of concordance lines for the term *opsec* in the/d/Laundromat corpus shows the term is typically used to refer to the technological measures taken to protect sensitive information and maintain privacy in operational activities. Its presence as a keyword offers further evidence of forum users’ concerns around security and anonymity. The only adjective on the list, *legit*, highlights legitimacy as an important issue to/d/Laundromat users and concordance lines featuring this term show that the concept is discussed in relation to businesses, individuals, funds, vendors, and websites, echoing findings regarding dark web fraud communities [50]. Its status as a keyword suggests that legitimacy of these entities is not taken for granted, but something that must be established.

The wordlist and keywords analyses have identified, in response to RQ1, that key discussion topics include finance and business, with a particular emphasis on cryptocurrencies, as well as methods and approaches to ML and fraud. The importance of computer security and the legitimacy of products and vendors was also highlighted. Lexical verbs in particular pointed to the primary function of this forum as a site for knowledge-exchange.

### Discursive practices of knowledge-exchange

A concordance analysis of the 20 most frequent 5-grams points to two primary discursive practices in the/d/Laundromat discourse. These are broadly defined as: 1. the giving of information, advice, or some other form of assistance, and 2. the seeking of information, advice or assistance (hereafter abbreviated to *information-giving* and *information-seeking*). This may be unsurprising for a forum specifically created as a site for community-based knowledge-exchange, but it is nonetheless notable that every 5-gram within the top 20 predominantly serves one or both of these functions, highlighting the narrowness of focus on ML as a central topic, and clearly delineating knowledge-exchange as the forum’s central purpose. It also reflects a general adherence by users to forum rules around staying on-topic and refraining from promoting services and posting spam messages. Indeed, arguably the closest a user gets to an off-topic contribution across the 263 concordance lines observed is offering a comment on the US series Ozark, a TV drama which centralises themes of fraud and money laundering.

The 20 most frequent 5-grams observed across the corpus can be organised into three broad functional categories; those that predominantly work towards giving information, those that predominantly work towards seeking information, and a single 5-gram that does both in roughly equal measure. It is worth noting that exceptions exist in each case and that the groupings merely indicate the *primary* function of each construction. The following section discusses the 5-grams within each category in terms of their individual discourse functions according to Biber and Barbieri’s [44] lexical bundle types. All textual examples are taken verbatim from the forum posts but may be clipped from longer contributions. Where examples include one of the 5-grams in question, these have been italicised.

### Information-giving

Of the 20 high-use 5-grams, 12 are predominantly used as part of information-giving contributions (see Table 3), arguably establishing information-giving as the most important and characteristic practice that occurs in this forum.

**Table 3:** 5-grams associated with information-giving.

5-gram	Raw frequency
*you don t want to*	22
*you don t have to*	21
*at the end of the*	20
*you don t need to*	16
*as far as i know*	11
*where the money came from*	11
*i don t know what*	11
*as long as you pay*	10
*if you don t have*	10
*i m not sure if*	9
*if you don t want*	9
*if you need any help*	9

The 5-grams most clearly associated with giving information, advice and assistance are those that express a stance of (often negative) obligation:

*you don’t want to* leave any mystique about your workings

*you don’t have to* pay tax on that money

*you don’t need to* do any actual work

youre fine *as long as you pay* taxes on it

Where these constructions contribute to clear, authoritative offers of information and advice, epistemic constructions expressing uncertainty are also fairly common in information-giving:

… inheritance is always exempted *as far as i know*…

… however *I’m not sure if* you need to put the real address

I havnt used a prepaid debt in awhile, so *I don’t know what* info they want…

As in the first example, the construction *as far as I know* is typically used as a hedging device to reduce the force of the user’s assertion, leaving room for the possibility that the information offered may be inaccurate or incomplete. In this way, this construction works to mitigate the authority of the user and limit potential for criticism from others. Comparatively, the constructions *I’m not sure if* and *I don’t know what* convey even less certainty, illustrating further that users can be forthcoming about gaps in their knowledge when offering advice and information. It is likely that both of these lower-certainty epistemic constructions in fact contribute to an overall impression of expertise, as they explicitly indicate users’ self-awareness regarding the limits of their knowledge.

Other information-giving 5-grams serve a discourse-organising function by refining the topic to address another user’s specific needs or desires.

… *if you don’t have* a guy for your monero buying then localmonero is really your only option

*If you don’t want* the transaction in crypto you need to do a swift transfer...

As these examples show, users often demonstrate a breadth of knowledge and experience by offering appropriate solutions for a range of needs and scenarios. Another discourse-organising construction is used simply to offer help in a general sense:

*If you need any help*, feel free to reach out

PM me *if you need any help* with the process, technicalities etc.

Explicit offers of help such as these are often accompanied by an invitation for the help-seeker to privately message the information-giver outside of the main forum discussion, highlighting both the open and cooperative nature of this community, and its primary purpose as a platform for knowledge-exchange.

Another 5-gram that predominantly contributes towards information-giving is *at the end of the*. The high use of this construction can be attributed to its dual functions; of the 17 instances that work towards information-giving, nine form part of the idiomatic phrase *at the end of the day* which serves to summarise and/or emphasise a piece of information offered:

treat it like regular business, because *at the end of the* day it is a business

… *at the end of the* day you got lucky in the NFT bubble;)

whereas eight instances relate directly to the proposition being discussed, which in most cases concerns the end of the financial year:

Only profits/losses need to be reported *at the end of the* year

… because *at the end of the* year the irs is still going to ask where…

The final 5-gram associated with information-giving does not fit neatly into Biber and Barbieri’s [44] bundle types, but rather points directly to the most fundamental task involved in ML, i.e., explaining *where the money came from*:

… you will need to be able to explain *where the money came from*

Obviously they cant prove *where the money came from*…

This construction tends to be used as either a part of general advice about the fundamentals of ML, or in reference to specified scenarios. The high use of this topic-specific 5-gram not only further demonstrates the narrowness of focus on the topic of ML, it reiterates the centrality of this particular narrative task that lies at the heart of all ML-related activities. Uses of this construction also show that while much of the information and advice given in this forum is highly technical, some users arrive with little understanding of what is involved in ML and seek very basic information.

### Information-seeking

Seven high-use 5-grams predominantly work towards the discursive practice of information-seeking (see Table 4).

**Table 4:** 5-grams associated with information-seeking.

5-gram	Raw frequency
*i don t want to*	21
*i don t know how*	16
*what is the best way*	13
*how would i go about*	11
*is the best way to*	11
*what would be the best*	11
*but i don t know*	11

Of the seven 5-grams associated with information-seeking, four contribute to direct questions about how to accomplish a particular ML-related task, and serve the discourse-organising function of focusing the topic onto the particular type of knowledge or assistance sought:

*what is the best way* to cash out 150k in xmr?

What *is the best way to* completely break the traceability of funds?

*What would be the best* way to buy BTC with cash?

*How would I go about* turning a.jpeg artwork into a saleable NFT?

Notably, the common use of the superlative adjective demonstrates that when seeking information, users often specify a request for ‘the best’ method available. This formulation explicitly acknowledges that there may be several approaches to solving a particular problem, potentially indexing a certain level of subject knowledge and technical competence.

The three remaining 5-grams work towards a less direct approach by which users implicitly invite others to offer solutions to their problems:

Ideally, *I don’t want to* be selling BTC for cash for the next decade

I recently found a Indian bank account and *I don’t know how* to cashout from it.

I know a little bit about deFi in ethereum *but I don’t know* of any specific strategy

As in the first example above, the 5-gram *I don’t want to* expresses a stance of desire and is largely used to show a preference (or dispreference) for a particular kind of approach or method to ML activities. Conversely, *I don’t know how* and *but I don’t know* express stances of uncertainty, plainly referring to gaps in the users’ knowledge. As the two latter examples illustrate, where users explicitly state gaps in their knowledge as a means of information-seeking, these statements are often accompanied or ‘balanced’ by contextual details that demonstrate a level of experience or understanding which may serve to make up for the knowledge deficit. This is another means by which information-seeking users index themselves as competent individuals, perhaps as a way of demonstrating their worthiness of other users’ time, effort and advice.

Finally, it is worth noting the 5-gram that works towards both information-seeking and giving in almost equal measure. The construction *to find a way to* has a discourse-organising function of narrowing the topic of discussion towards specific ML-focused goals and the methods required to achieve them:

I’m trying *to find a way to* get this money in circulation (info-seeking)

I will need *to find a way to* get the money from the CC… (info-seeking)

… you need *to find a way to* launder it (info-giving)

you either need *to find a way to* make multiple bank accounts… (info-giving)

That this dual-function 5-gram is of high use within the corpus further illustrates that the discourse in this forum is goal-centred, and that the methods and processes involved in ML-related activities are the central aspect of the information both sought and given in this forum.

Addressing RQ2, the 5-grams analysis has pointed to a number of linguistic constructions associated with giving and seeking advice and/or assistance. These constructions have pointed in various ways to the community’s commitment to focusing on the central topic of ML, the broad expertise of community members (whether seeking or giving advice), and to a general magnanimous approach to sharing information.

## Discussion and conclusions

The analysis has shown that/d/Laundromat forum discussions typically centralise practical issues around the methods and processes involved in ML-related activities, which reflects its overall purpose as a community-based platform for knowledge exchange. Frequent words and keywords pointed to prominent discussion topics including those which are expected and confirmatory (e.g., business, finance, fraud, security) and those that offer genuine new insight about a community that has formed around an interest in ML, such as cryptocurrencies and the perceived legitimacy of various entities including individuals, vendors and funds. The topic of cryptocurrencies features particularly heavily despite the observation that cash remains the preferred commodity in ML schemes due to its comparable stability and practicality [1,26]. This is likely in part a function of this particular online context; within the wider ecology of dark-web fora that exist around various criminal pursuits (e.g., illicit marketplaces, fraud fora), including Dread with its 1,750 subfora, cryptocurrencies have proven extremely popular as a commodity due to their untraceability, and as such, are widely discussed. Arguably it would be surprising if this interest did not carry over to a forum for knowledge-exchange around money laundering. It may also indicate a shift towards an increased use of cryptocurrencies within laundering processes; as technologies continually evolve, so too do the tools and methods available for internet-enabled crimes. Alternatively, as a learning platform, /d/Laundromat may overrepresent less experienced individuals who lack understanding about the practicalities of using cryptocurrencies in relation to ML but are particularly interested in their applicability in this context.

Notably, the community does not tend to discuss potential moral implications of their activities, or exchange emotional support; two practices often observed among criminal communities that form around more emotive and distressing types of crime such as CSEA (see [33,43,51]). It may be that ML is less psychologically troubling than other forms of criminal activity, especially for those who launder money for others and in so doing remain a step removed from the predicate crimes that feed into it (e.g., human trafficking, illegal arms trading), ensuring a certain distance from the actual harm suffered by victims. Philosophical discussion around the concept of ML as a ‘victimless crime’ as based on Mill’s Harm Principle [52] sees debate regarding the criminalisation of ML versus other potential approaches to dealing with the proceeds of crime (e.g., confiscation and forfeiture) ([53–55]), and further complicates the perceived moral implications of ML. A central issue in this debate concerns whether laundering is considered a form of complicity in predicate crimes [53]. While discussion around predicate crimes themselves do occur in the/d/Laundromat corpus, they are by no means prominent topics. Combined with the lack of discussion around morality or the possible psychological needs of users, this demonstrates that ML is discursively treated in/d/Laundromat as distinct and somewhat abstracted from the predicate crimes that might provoke stronger moral concern.

Biber and Barbieri’s [44] bundle types have provided a useful approach to understanding the discourse functions of the common 5-grams used by/d/Laundromat users. Notably, and in response to RQ3 (To what extent does this community of practice serve the needs of its members and how does it do this?), we have seen that every one of the 20 most common constructions contributed towards an overall function of either giving or seeking information and advice around money laundering, pointing to/d/Laundromat as an extremely efficient and productive site for knowledge-exchange. The effectiveness of knowledge-exchange within this community can be attributed to an interplay of at least three components:

1. Adherence to community rules. The general observance of rules around remaining on-topic and refraining from the promotion of personal services ensures that information-seeking/d/Laundromat users are not required to sift through numerous posts containing irrelevant content or advertisements (common to some fraud-focused fora) in order to access genuinely sought-after information. The requirement to stay on-topic also makes the discourse of this community more transactional and less of a social activity than in other communities for whom social interaction appears as important as the primary activity or issue that convenes them, e.g., the exchange of indecent images of children [34] far-right extremism [35] or ‘paedophile-hunting’ [51]
2. A highly knowledgeable user base. Many users demonstrate expertise across a broad range of ML-related activities and issues, and offer this expertise in a straightforward communicative style while showing self-awareness around knowledge gaps and limitations. Furthermore, information-seeking was often seen to involve subtle expressions of domain-specific competence, which likely have a persuasive function of demonstrating that those seeking help are serious members of the community, often with experience in other areas of ML, and most importantly, worthy of help. It is worth remembering that new methods and approaches to fraud and ML are presented all the time, meaning that seasoned experts have equal cause to newcomers for seeking assistance.
3. A culture of friendliness and reciprocity. On the whole, users will readily help each other, offering advice at a range of levels from highly technical information about specific methods and processes to basic explanations around what it means to launder money. This general openness to support others reflects a community-wide understanding of the purpose of the forum to operate as a dynamic, reciprocal informational resource, as well as the success of the community in meeting this aim.

This work has identified common topics and discursive practices associated with knowledge exchange in a dark-web forum focused on money laundering. In doing so, it contributes an empirically based linguistic perspective to the limited work addressing the social and behavioural aspects of money laundering. As a community of practice, /d/Laundromat has been shown to offer its users learning support across a diverse range of ML-related issues for users of varied levels of domain-specific competence. The three key aspects characterising this community – a strict adherence to forum rules, a highly knowledgeable user base, and a culture of friendliness and openness – each contribute to its success as an effective and productive platform for knowledge-exchange, unclouded by promotional posts and less impacted by the socially oriented discourse and moral concerns that often pervade other types of deviant online fora. Importantly, these characteristics make/d/Laundromat a socially safe space for newcomers to begin learning the fundamentals of money laundering, and a fruitful site for the continued development of that knowledge and expertise over time. In this way, the discourse of the/d/Laundromat community provides an illustrative example of how dark-web knowledge-exchange platforms can become key facilitators of criminal upskilling and enablers of continued criminal activity. Future work examining several ML-focused online spaces could offer comparisons between groups, providing a more comprehensive and nuanced picture of offending and behaviour around money laundering.
